# Temperature-Responsive Photoluminescence and Elastic Properties of 1D Lead Halide Perovskites *R*- and *S*-(Methylbenzylamine)PbBr_3_

**DOI:** 10.3390/molecules27030728

**Published:** 2022-01-23

**Authors:** Rui Feng, Jia-Hui Fan, Kai Li, Zhi-Gang Li, Yan Qin, Zi-Ying Li, Wei Li, Xian-He Bu

**Affiliations:** 1 College of Chemistry & State Key Lab of Elemento-Organic Chemistry, Nankai University, Tianjin 300071, China; fengrui1226@hotmail.com (R.F.); buxh@nankai.edu.cn (X.-H.B.); 2 School of Materials Science and Engineering & Tianjin Key Laboratory of Metal and Molecule-Based Material Chemistry, Nankai University, Tianjin 300350, China; asfjhh@163.com (J.-H.F.); 1120180353@mail.nankai.edu.cn (K.L.); 1120200436@mail.nankai.edu.cn (Z.-G.L.); 1120210484@mail.nankai.edu.cn (Z.-Y.L.); 3 School of Physics & Wuhan National Laboratory for Optoelectronics, Huazhong University of Science and Technology, Wuhan 430074, China; qinyan@hust.edu.cn

**Keywords:** low-dimensional, metal halide perovskite, photoluminescence, stimulus-responsive, elastic property

## Abstract

Low-dimensional metal halide perovskites (MHPs) have received much attention due to their striking semiconducting properties tunable at a molecular level, which hold great potential in the development of next-generation optoelectronic devices. However, the insufficient understanding of their stimulus-responsiveness and elastic properties hinders future practical applications. Here, the thermally responsive emissions and elastic properties of one-dimensional lead halide perovskites *R*- and *S*-MBAPbBr_3_ (MBA^+^ = methylbenzylamine) were systematically investigated via temperature-dependent photoluminescence (PL) experiments and first-principles calculations. The PL peak positions of both perovskites were redshifted by about 20 nm, and the corresponding full width at half maximum was reduced by about 40 nm, from ambient temperature to about 150 K. This kind of temperature-responsive self-trapped exciton emission could be attributed to the synergistic effect of electron–phonon coupling and thermal expansion due to the alteration of hydrogen bonding. Moreover, the elastic properties of *S*-MBAPbBr_3_ were calculated using density functional theory, revealing that its Young’s and shear moduli are in the range of 6.5–33.2 and 2.8–19.5 GPa, respectively, even smaller than those of two-dimensional MHPs. Our work demonstrates that the temperature-responsive emissions and low elastic moduli of these 1D MHPs could find use in flexible devices.

## 1. Introduction

Metal halide perovskites (MHPs) are attracting considerable interest owing to their excellent optoelectronic properties tunable at a molecular level [1,2,3,4,5]. The merits of a high absorption coefficient, good defect resistance, and ease of synthesis [6,7,8] have led to their wide application in solar cells [9,10,11,12], photodetectors [13,14,15], and light-emitting diodes [16,17,18,19,20,21]. Currently, the number of reported three-dimensional (3D) MHPs is very limited due to their structural requirement by the Goldschmidt tolerance factor [6,7,8]. To overcome this restriction, low-dimensional (LD) MHPs, including zero-dimensional (0D), one-dimensional (1D), and two-dimensional (2D) MHPs, are being widely explored. In comparison to their 3D counterparts, LD-MHPs possess higher environmental and thermal stability, as well as larger chemical and structural diversity [22,23,24]. Accordingly, these LD-MHPs have received intense attention in both synthesis studies and applications [25,26].

In these LD-MHPs, the distortion of PbX_6_ octahedra (X = halogen) significantly influences their photoluminescence (PL) behaviors. Hydrogen bonding, as one of the widely available interactions connecting the inorganic and organic parts, plays an important role in determining the magnitude of octahedral distortion [27,28]. By changing strengths of hydrogen bonds upon external stimuli (i.e., temperature and pressure), the emissive processes and properties of LD-MHPs, such as peak position, intensity, and the full width at half maximum (FWHM) of self-trapped excitons (STEs), could be manipulated [29,30,31,32]. Although there have been a handful of reports about the influence of hydrogen bonding on the PL properties of LD-MHPs upon external stimulation, more efforts should be devoted to elucidating the underlying mechanism. In addition, the elastic properties of materials are of vital importance since they not only determine the long-term reliability and endurance in service but also regulate the manufacturing and processing [33,34]. However, very little attention has been paid to the understanding of the elastic properties of LD-MHPs [35,36].

In this work, the temperature-responsive PL of a pair of 1D MHPs, *R*- and *S*-MBAPbBr_3_, was systematically investigated by variable-temperature optical spectroscopy. Our results indicate that both perovskites exhibit typical yellow emission under ambient conditions ascribed to the STE emission. Their emission peaks show a remarkable redshift and a significant enhancement of intensity with decreasing temperature. In addition, the elastic properties of *S*-MBAPbBr_3_ were comprehensively studied via density functional theory (DFT) calculations.

## 2. Results and Discussion

### 2.1. Crystal Structures

**Figure 1:**
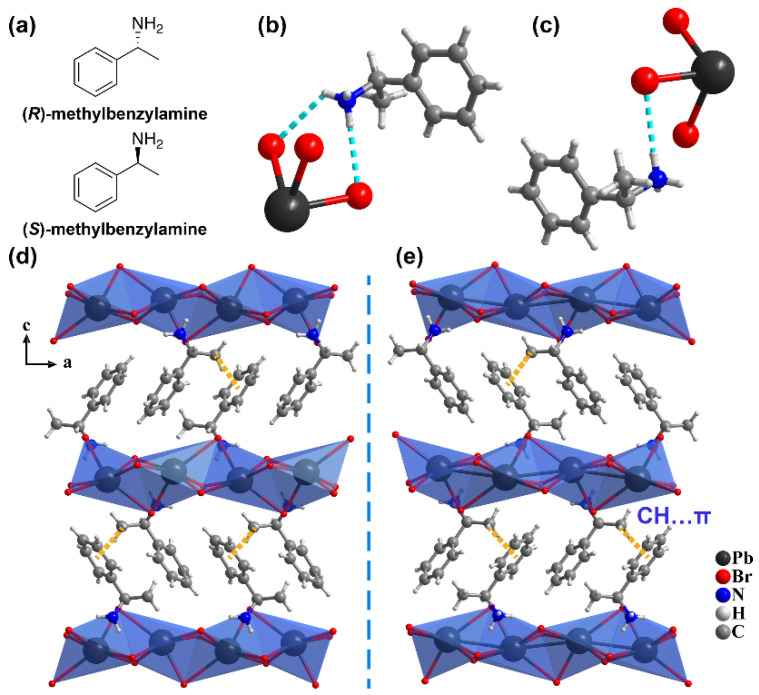
The structure of MBAPbBr_3_. (**a**) The molecular structure scheme of methylbenzylamine. (**b**,**c**) Hydrogen bonds between the [PbBr_3_]^−^ chain and methylbenzylamine in MBAPbBr_3_. (**d**,**e**) The structures of *R*-MBAPbBr_3_ (**d**) and *S*-MBAPbBr_3_ (**e**) along the *b*-axis.

Both *R*- and *S*-MBAPbBr_3_ crystallize in the chiral *P*2_1_2_1_2_1_ space group, which is consistent with reports in the literature [37]. Taking *S*-MBAPbBr_3_ as an example, its cell parameters at 100 K are *a* = 7.8835(3) Å, *b* = 8.0680(3) Å, and *c* = 20.1237(8) Å. The asymmetric unit of the structure consists of a methylbenzylamine cation and a [PbBr_3_]^−^ unit (Figure 1c). The six-coordinated Pb atoms are coordinated by six Br atoms to form a PbBr_6_^−^ octahedron, and adjacent PbBr_6_ octahedra are face-shared to form an infinite inorganic chain along the *a*-axis. Each inorganic chain interacts with surrounding organic amine cations via electrostatic forces and N–H···Br hydrogen bonding in a hexagonal manner, forming a 1D organic–inorganic assembly with a chemical formula of *S*-MBAPbBr_3_ (Figure 1e). Adjacent 1D organic–inorganic assemblies are connected by intermolecular CH…π interactions with distances of 3.383 Å, giving rise to a 3D supramolecular structure. To evaluate the structural change upon temperature, the structure was collected at 293 K and compared with that at 100 K. Specifically, the lengths of Pb–Br bonds of *S*-MBAPbBr_3_ are in the range of 2.857–3.062 Å and 2.852–3.070 Å at 100 and 293 K, respectively. The distances between N and Br atoms in N–H···Br hydrogen bonds are 3.387–3.499 Å and 3.428–3.558 Å at 100 and 293 K. As mentioned above, hydrogen bonding plays an important role in the octahedral distortion degree. As shown in the distance of N–Br (Table S1), the hydrogen bonding becomes stronger at lower temperature, causing distinct octahedral distortion in the *c*-direction. With the temperature increase, the increased vibrations of MBA molecules weaken the hydrogen bonding, thus reducing the distortion degree, and *S*-MBAPbBr_3_ expands in the *c*-direction. Combined with the cell parameters at 100 and 293 K of *S*-MBAPbBr_3_ (Table S1, Figure S1), the *c*-axis shows the highest coefficient of thermal expansion, which is consistent with the above analysis, indicating that the distortion of inorganic chains can be adjusted by varied hydrogen bonding upon thermal stimulus. The degree of [PbBr_6_] distortion can be quantified by the mean octahedral quadratic elongation (λ) and variance of the octahedral angle parameters ($\sigma^{2}$), defined as follows [38]:

(1) $$\lambda =\frac{1}{6}\overset{6}{\underset{i=1}{\sum }}{\left(d_{i}/d_{0}\right)}^{2},$$

(2) $$\sigma^{2}=\frac{1}{11}\overset{12}{\underset{i=1}{\sum }}{\left(\alpha_{i}-90\right)}^{2},$$

where $d_{i}$ denotes the six individual bond lengths of Pb–Br, $d_{0}$ denotes the average distance of the bond length of Pb–Br, and $\alpha_{i}$ denotes the individual bond angle of Br–Pb–Br. The calculated λ and $\sigma^{2}$ for *S*-MBAPbBr_3_ are 1.003 and 221.58, and 1.003 and 197.04, at 100 and 293 K, respectively. The above results suggest that the distortion of octahedra is mainly manifested as the change of bond angles, and the structure at lower temperature is more distorted due to the alteration of hydrogen bonds. This could lead to temperature-responsive emission, as we discuss below.

### 2.2. Electronic Structures

To investigate the electron structural properties, the electronic band structures and density of states of both *R*- and *S*-MBAPbBr_3_ were calculated via DFT (Figure S2); the two structures have almost identical electronic band structures. The valence band maximum (VBM) and conduction band minimum (CBM) of *R*- and *S*-MBAPbBr_3_ are located at (0.236842, 0.5, 0.5) and (0, 0, 0) in *k*-space, showing indirect bandgaps of 3.571 and 3.573 eV, respectively. The partial density of states was subsequently calculated to identify the orbital contribution during the excitation process. The VBMs of *R*- and *S*-MBAPbBr_3_ are mainly contributed by the 4*p* orbital of Br atoms, and the two CBMs are mainly derived from the 6*p* orbital of Pb atoms. The above results indicate that the band edges of the two perovskites are mainly contributed by inorganic PbBr_6_ octahedra [39].

### 2.3. PXRD and TGA Measurements

The phase purities of both *R*- and *S*-MBAPbBr_3_ were confirmed by powder X-ray diffraction (PXRD). The cell parameters of the observed crystal were refined with the TOPAS-v6 software using a Le Bail algorithm (Figure S3). The peak positions of both *R*- and *S*-MBAPbBr_3_ are almost the same, and the variant peak intensity can be attributed to the difference of exposed crystal surface after grinding. The TGA curves show a plateau below 225 °C and a weight loss of 35.5% between 225 and 230 °C, identifying their stability (Figure S4). The mass loss near 230 °C can be attributed to the removal of vaporization of methylbenzylamine (21.3%) and HBr (14.2%). The good stability of MBAPbBr_3_ warrants its further characterization.

### 2.4. Optical Properties

UV–Vis absorption spectra were determined to characterize the excitation behavior (Figure S7). The absorptions of the two 1D MHPs are almost identical as expected for enantiomeric structures, with exciton absorption peaks at 330 nm. The diffuse reflectance measurements were converted to the Kubelka–Munk method, and the bandgaps were calculated using the Kubelka–Munk function $F\left(R\right)={\left(1-R\right)}^{2}/2R$, where R represents the reflection coefficient. The bandgaps for *R*- and *S*-MBAPbBr_3_ were estimated to be 3.59 eV and 3.67 eV, respectively, which are consistent with the calculated values of about 3.57 eV from DFT.

**Figure 2:**
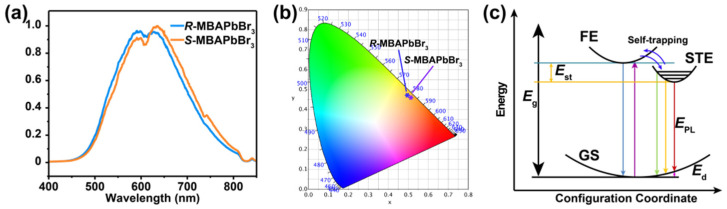
The PL properties of *R*- and *S*-MBAPbBr_3_. (**a**) PL spectra at 296 K excited by a 325 nm laser. (**b**) The CIE coordinates of PL. (**c**) The configuration coordinate models of PL. FE: free exciton, GS: ground state, *E*_g_: bandgap, *E*_st_: self-trapped energy, *E*_d_: lattice distortion energy, *E*_PL_: emission energy.

Under irradiation with UV light, the crystals of *R*- and *S*-MBAPbBr_3_ show yellow emission at room temperature (Figure 2a). Both perovskites have two broad emission peaks extending across the cyan color to the near-infrared region. The maximum emission wavelengths of *R*- and S-MBAPbBr_3_ are 594 and 616 nm, and 592 and 618 nm, respectively. The FWHMs of *R*- and *S*-MBAPbBr_3_ are estimated to be 181.3 and 178.1 nm, respectively. The appearance of two emission peaks may be attributed to two kinds of exciton paths. To illustrate the PL color at room temperature clearly, the Commission Internationale de L’Eclairage (CIE) chromaticity diagram and color temperatures of PL are illustrated in Figure 2b. The CIE coordinates of *R*- and *S*-MBAPbBr_3_ are (0.498, 0.471) and (0.510, 0.463) with the color temperatures of 2647 and 2470 K, respectively.

The diagram of the PL process is shown in Figure 2c. Upon UV light irradiation, the electrons in the ground state are excited to form free excitons. Some free excitons radiate photons and return to the ground state directly, which is known as free-exciton emission. Due to lattice distortion caused by strong electron–phonon coupling, some excitons become self-trapped, emitting photons with reduced energy before returning to the ground state [40]. This STE radiative process leads to the broad emission spectra of the two 1D MHPs.

**Figure 3:**
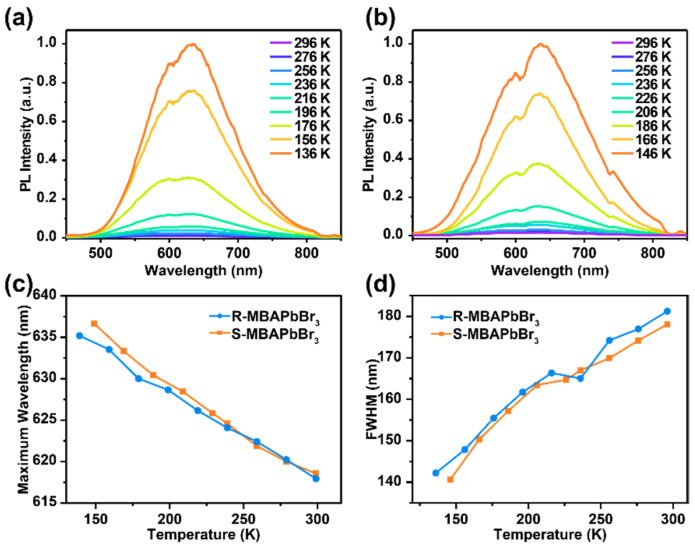
(**a**,**b**) The PL spectra of *R*-MBAPbBr_3_ (**a**) and *S*-MBAPbBr_3_ (**b**) at various temperatures. (**c**) The maximum wavelengths of *R*- and *S*-MBAPbBr_3_ at different temperatures. (**d**) The FWHMs of *R*- and *S*-MBAPbBr_3_ at different temperatures.

To further explore the properties of the STE emission behavior, PL spectra at various temperatures were collected (Figure 3). As the temperature decreases from 296 to 146 K, the broad emission peaks gradually redshift by approximately 20 nm with decreased FWHM from 181.3 to 142.2 nm for *R*-MBAPbBr_3_ and 178.1 to 140.6 nm for *S*-MBAPbBr_3_, respectively. It is interesting that the PL intensity is increased by about two orders of magnitude with the reduction in temperature. The variation in FWHM could arise from the synergistic effect of electron–phonon coupling and thermal expansion, which is influenced by the strength change of hydrogen bonding. The higher intensity and narrower peak width at low temperatures can be attributed to the suppression of nonradiative complexation of excitons [41,42,43].

### 2.5. Elastic Properties

**Figure 4:**
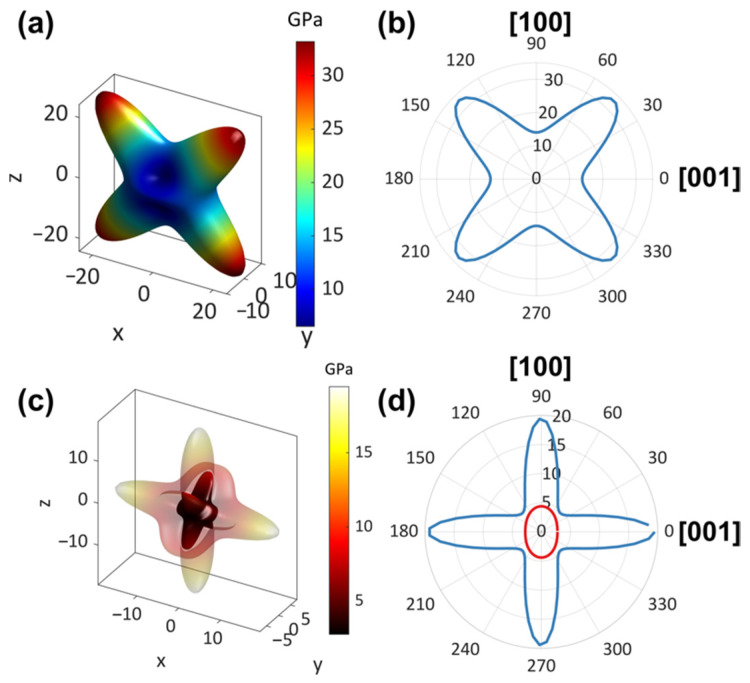
3D and 2D representations of Young’s moduli (**a**,**b**) and shear moduli (**c**,**d**) of *S*-MBAPbBr_3_. In (**c**), the transparent outer layer and the nontransparent inner layer denote the maximum and minimum values, respectively. The blue outer line and red inner line in (**d**) denote the maximum and minimum values, respectively.

To investigate the elastic properties, the elastic constants (*C*_ij_) and bulk modulus (*K*) of *S*-MBAPbBr_3_ were calculated by DFT, and the obtained results are listed in Table S7. According to its *C*_ij_, the maximal and minimal values of Young’s moduli (*E*) and shear moduli (*G*) were extracted using the ELATE software [44] as presented in Table S7. The representative 3D and 2D plots of *E* are shown in Figure 4a,b. The maximum value of *E* (*E*_max_) for this perovskite is 33.2 GPa along the <101> direction due to the large Br–Pb–Br bond angle (154.9°) in this direction. In addition, its *E* reaches the minimum value (*E*_min_) of 6.5 GPa along the <011> direction, which could be attributed to the compliant nature of organic cations packing along this orientation. Accordingly, these two values give an elastic anisotropy (A*_E_* = *E*_max_/*E*_min_) of 5.1, which is relatively larger than that of some 2D MHPs, such as (benzylammonium)_2_PbBr_4_ (4.9) [45] and (4-methoxyphenethyammonium)_2_PbI_4_ (3.2) [46]. Moreover, the extracted 3D and 2D plots of *G* for *S*-MBAPbBr_3_ are shown in Figure 4c,d. It can be observed that the maximal *G* (*G*_max_) is 19.5 GPa along the <010> direction when the (001) plane is sheared, which can be ascribed to the rigid [PbBr_3_]^−^ inorganic chains that can significantly resist deformation under the shear force. However, the minimal *G* (*G*_min_) of 2.8 GPa occurs along the <100> inorganic chain direction when the same plane is sheared, which arises from the facile sliding of the 1D inorganic chains under shearing. The obtained elastic anisotropy (A*_G_* = *G*_max_/*G*_min_) of *S*-MBAPbBr_3_ is 7.0, which is larger than that of 2D (benzylammonium)_2_PbBr_4_ (6.5) and (4-methoxyphenethyammonium)_2_PbI_4_ (4.0).

The calculated *K* of *S*-MBAPbBr_3_ is 7.3 GPa, which is significantly smaller than the reported values of 2D MHP (benzylammonium)_2_PbBr_4_ (13.6 GPa) and (4-methoxyphenethyammonium)_2_PbI_4_ (9.8 GPa), indicating that *S*-MBAPbBr_3_ with a 1D structure is more prone to hydrostatic deformation compared with 2D MHPs. According to Pugh’s criterion [47], the brittleness of materials can be quantified by the ratio of *K*/*G*. The materials with *K*/*G* < 1.75 are called brittle. The *K*/*G* ratio of *S*-MBAPbBr_3_ in the range of 0.17–1.99, implying that this MHP would be fairly brittle along certain directions. The low elastic modulus of *S*-MBAPbBr_3_ implies that these 1D MHPs could be more desirable for applications in flexible devices, in comparison to 2D and 3D MHPs, although their fragile nature along certain crystallographic directions needs to be taken into account.

## 3. Materials and Methods

The synthetic method of chiral *R*-MBAPbBr_3_ is described in the literature [37,39]. (*R*)-Methylbenzylamine (C_8_H_11_N, 0.15 g, 1 mmol, Figure 1a) and lead bromide (PbBr_2_, 0.239 g, 0.5 mmol) were added to a mixture of acetonitrile (5 mL) and hydrobromic acid (HBr, 5 mL) in a beaker. The mixture was stirred and sonicated to obtain a colorless solution, and the solution was slowly evaporated overnight. The colorless crystal was washed with methanol and dried under vacuum (melting point: 208 °C). The synthetic method of chiral *S*-MBAPbBr_3_ is similar to that of *R*-MBAPbBr_3_ except (*R*)-methylbenzylamine was replaced by (*S*)-methylbenzylamine. Melting point: 209 °C. The mass spectra of *R*- and *S*-MBAPbBr_3_ are shown in Figures S5 and S6.

The single-crystal X-ray diffraction (SC-XRD) tests of *S*-MBAPbBr_3_ were performed using a Rigaku XtaLAB PPO MM007 CCD diffractometer with a Cu-K*α* target radiation source (λ = 1.54184 Å) at 293 K and MoK*α* (λ = 0.71073 Å) at 100 K, respectively. Using Olex2 [48], the structure was directly solved by ShelXT [49] and refined anisotropically for all nonhydrogen atoms by full-matrix least squares on all F^2^ data using ShelXL [50]. All hydrogen atoms were added according to the theoretical model with isotropic displacement parameters and allowed to ride on parent atoms.

Powder X-ray diffraction (PXRD) tests were performed using a Rigaku MiniFlex 600 diffractometer. The samples of *R*- and *S*-MBAPbBr_3_ were tested in the range of 3–50° with a step size of 0.02° and a speed of 3°·min^−1^.

Thermogravimetric analysis (TGA) was performed using a Thermo plus EVO2 TG-DTA 9121 thermoanalyzer under N_2_ atmosphere with a flow rate of 50 mL·min^−1^. The measurement temperature ranged from 25 °C to 800 °C with a change rate of 10 °C·min^−1^.

The electronic structure was calculated taking the generalized gradient approximation with a Perdew–Burke–Ernzerh (GGA-PBE) exchange-correlation functional [51] by VASP [52,53,54]. The plane-wave cutoff energy was set to 450 eV, and a Monkhorst–Pack K-point sampling of 3 × 3 × 1 was used to sample the Brillouin zone. During the geometry optimization step, the cell parameters and atom positions were fully relaxed. The total energy and residual force on each atom converged to 10^−6^ eV and 0.01 eV⋅Å^−1^, respectively. The elastic stiffness constants *C*_ij_ were obtained by the stress–strain method with 0.015 Å of the maximum strain amplitude and seven steps for each strain.

The UV–Vis spectra were measured using a Solidspec 3700 UV–Vis–NIR spectrophotometer with a standard reference of BaSO_4_ at room temperature. The wavelength range was set to 200–800 nm. Variable temperature photoluminescence experiments were performed using a Horiba LabRAM HR 800 Raman spectrometer excited by a 325 nm He–Cd laser. The photoluminescence (PL) spectra were dispersed by a 600 groove per millimeter diffraction grating and accumulated two times with 2 s of exposure.

## 4. Conclusions

In summary, the temperature-responsive PL properties and elastic properties of 1D MHPs, *R*- and *S*-MBAPbBr_3_, were systematically investigated via combined experimental and theoretical approaches. Both *R*- and *S*-MBAPbBr_3_ exhibit yellow emissions covering a wide wavelength range. With decreasing temperature, the STE emission peaks of both perovskites exhibit narrowed widths and redshifted positions. In addition, the temperature reduction leads to an intensity enhancement of about two orders of magnitude, which can be ascribed to the synergistic effect of electron–phonon coupling and thermal expansion influenced by the alteration of hydrogen bonding. In addition, our DFT calculations reveal that *S*-MBAPbBr_3_ exhibits a relatively large elastic anisotropy and small bulk modulus, compared with 2D and 3D MHPs. This work demonstrates the temperature-responsive emissions and low elastic properties of LD-MHPs could be useful for making smart optoelectronic devices.

## Data Availability

The crystal data of *S*-MBAPbBr_3_ are deposited in The Cambridge Crystallographic Data Centre (Nos. 2132892–2132893).

## Supplementary Materials

The following supporting information can be downloaded online: Table S1. The cell parameters of *S*-MBAPbBr_3_ at different temperatures; Figure S1. The change of cell parameters of *S*-MBAPbBr_3_ at different temperatures and the diagram of thermal expansion; Table S2. The crystal data and structure refinement for *S*-MBAPbBr_3_ at 100 K and 293 K; Table S3. Bond lengths for *S*-MBAPbBr_3_-100 K; Table S4. Bond angles for *S*-MBAPbBr_3_-100 K; Table S5. Bond lengths for *S*-MBAPbBr_3_-293 K; Table S6. Bond angles for *S*-MBAPbBr_3_-293 K; Figure S2. The electronic structures of *R*- and *S*-MBAPbBr_3_; Figure S3. The PXRD fitting of MBAPbBr_3_; Figure S4. The TGA curves of *R*- and *S*-MBAPbBr_3_; Figure S5. The mass spectrum of *R*-MBAPbBr_3_; Figure S6. The mass spectrum of *S*-MBAPbBr_3_; Figure S7. The UV–Vis absorption spectra of *R*- and *S*-MBAPbBr_3_; Table S7. Summary of the elastic properties of S-MBAPbBr_3_.

## Sample Availability

Samples of the compounds *R*- and *S*- MBAPbBr_3_ are available from the authors.
