# Supplementary material for: Temperature-Responsive Photoluminescence and Elastic Properties of 1D Lead Halide Perovskites R- and S-(Methylbenzylamine)PbBr3
Source: Molecules. 2022 Jan 23;27(3):728. doi: 10.3390/molecules27030728 (PMC8839927; doi:10.3390/molecules27030728)
Supplement: Supplementary file 1 [file molecules-27-00728-s001.zip › molecules-1538383-supplementary/Supporting information.pdf]

Supporting Information

Temperature-Responsive Photoluminescence and Elastic Properties of 1D Lead Halide Perovskites *R*- and *S*-(methylbenzylamine)$PbBr_3$

Rui Feng [1], Jia-Hui Fan [2], Kai Li [2], Zhi-Gang Li [2], Yan Qin [3], Zi-Ying Li [2], Wei Li [2,*] and Xian-He Bu [1,2]

[1] College of Chemistry & State Key Lab of Elemento-Organic Chemistry, Nankai University, Tianjin 300071, China; fengrui1226@hotmail.com (R.F.); buxh@nankai.edu.cn (X.-H.B.)
[2] School of Materials Science and Engineering & Tianjin Key Laboratory of Metal and Molecule-Based Material Chemistry, Nankai University, Tianjin 300350, China; asfjhh@163.com (J.-H.F.); 1120180353@mail.nankai.edu.cn (K.L.); 1120200436@mail.nankai.edu.cn (Z.-G.L.); 1120210484@mail.nankai.edu.cn (Z.-Y.L.)
[3] School of Physics & Wuhan National Laboratory for Optoelectronics, Huazhong University of Science and Technology, Wuhan 430074, China; qinyan@hust.edu.cn
* Correspondence: wl276@nankai.edu.cn

Table S1. The cell parameters of *S*-$MBAPbBr_3$ at different temperatures.

T (*K*)	*a* (Å)	*b* (Å)	*c* (Å)	*α*=*β*=*γ* (°)	*V* (Å^3)
100	7.883	8.074	20.126	90	1281.075
120	7.887	8.080	20.157	90	1284.613
140	7.890	8.085	20.190	90	1288.264
160	7.893	8.094	20.221	90	1291.942
180	7.897	8.100	20.262	90	1296.284
200	7.901	8.108	20.303	90	1300.806
220	7.905	8.114	20.349	90	1305.391
240	7.910	8.120	20.399	90	1310.404
260	7.917	8.125	20.435	90	1314.566

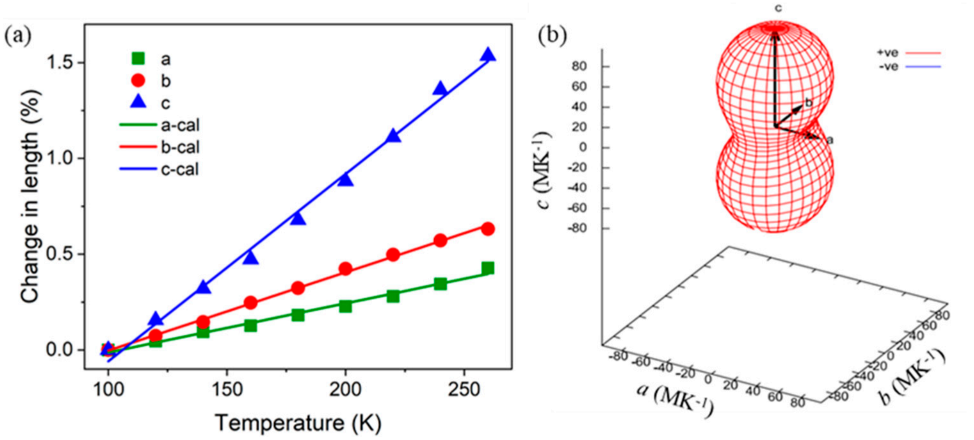

Figure S1. The change of cell parameters of *S*-$MBAPbBr_3$ at different temperatures (a) and the diagram of thermal expansion (b).

Table S2. The crystal data and structure refinement for *S*-$MBAPbBr_3$ at 100 K and 293 K.

Identification code	*S*-$MBAPbBr_3$-100 K	*S*-$MBAPbBr_3$-293 K
Empirical formula	$C_8H_{12}Br_3NPb$	$C_8H_{12}Br_3NPb$
Formula weight	569.11	569.11
Temperature/K	100.00(10)	293(2)
Crystal system	Orthorhombic	orthorhombic
Space group	$P2_12_12_1$	$P2_12_12_1$
a/Å	7.8835(3)	7.91780(10)
b/Å	8.0680(3)	8.13630(10)
c/Å	20.1237(8)	20.5574(3)
Volume/Å^3	1279.95(9)	1324.34(3)
Z	4	4
ρ_{calc} g/cm^3	2.953	2.854
μ/mm^{-1}	22.507	35.067
F(000)	1016.0	1016.0
Crystal size/mm^3	30 × 35 × 70	30 × 35 × 70
Radiation	MoK*α* (λ = 0.71073 Å)	CuK*α* (λ = 1.54184 Å)
2Θ range for data	7.226 to 50.044	8.602 to 133.102

collection/°		
Index ranges	$-9 \leq h \leq 9$, $-9 \leq k \leq 9$, $-23 \leq l \leq 23$	$-8 \leq h \leq 9$, $-9 \leq k \leq 9$, $-24 \leq l \leq 23$
Reflections collected	5033	5348
Independent reflections	2166 [R_{int} = 0.0524, R_{sigma} = 0.0491]	2343 [R_{int} = 0.0493, R_{sigma} = 0.0529]
Data/restraints/parameters	2166/0/121	2343/0/121
Goodness-of-fit on F^2	1.041	1.021
Final R indexes [I>=2σ (I)]	R_1 = 0.0449, wR_2 = 0.1061	R1 = 0.0410, wR2 = 0.1055
Final R indexes [all data]	R_1 = 0.0467, wR_2 = 0.1071	R1 = 0.0416, wR2 = 0.1061
Largest diff. peak/hole / e $Å^{-3}$	3.07/-1.59	3.29/-1.26
Flack parameter	0.01(2)	-0.006(11)

Table S3. Bond lengths for *S*-MBAPbBr$_3$-100 K.

Atom	Atom	Length/Å	Atom	Atom	Length/Å
Pb1	Br1	2.8835(16)	C4	C2	1.40(2)
Pb1	Br3	2.8565(16)	C4	C3	1.51(2)
Pb1	Br2^1	2.9690(16)	C4	C7	1.40(2)
Pb1	Br2	3.0617(16)	C6	C8	1.38(2)
Br2	Pb1^2	2.9690(16)	C6	C2	1.40(2)
N1	C3	1.51(2)	C8	C5	1.37(2)
C9	C3	1.51(2)	C5	C7	1.41(2)

^{1}1/2+X,3/2-Y,1-Z; ^{2}-1/2+X,3/2-Y,1-Z

Table S4. Bond angles for *S*-MBAPbBr$_3$-100 K.

Atom	Atom	Atom	Angle/°	Atom	Atom	Atom	Angle/°
Br1	Pb1	Br2	87.44(4)	C7	C4	C3	120.9(14)
Br1	Pb1	Br2^1	93.94(4)	C8	C6	C2	118.9(16)
Br3	Pb1	Br1	87.64(5)	C5	C8	C6	121.9(16)
Br3	Pb1	Br2^1	83.70(4)	C4	C2	C6	120.4(15)
Br3	Pb1	Br2	82.26(4)	C8	C5	C7	120.0(15)
Br2^1	Pb1	Br2	165.82(4)	N1	C3	C9	109.0(14)
Pb1^2	Br2	Pb1	82.77(4)	N1	C3	C4	108.6(13)
C2	C4	C3	119.1(14)	C9	C3	C4	115.7(15)
C2	C4	C7	119.9(15)	C4	C7	C5	118.9(15)

^{1}1/2+X,3/2-Y,1-Z; ^{2}-1/2+X,3/2-Y,1-Z

Table S5. Bond lengths for *S*-MBAPbBr$_3$-293 K.

Atom	Atom	Length/Å	Atom	Atom	Length/Å
Pb1	Br3	2.8939(14)	C3	C4	1.37(2)
Pb1	Br2	2.8521(14)	C3	C8	1.37(2)
Pb1	Br1	2.9861(14)	C2	C1	1.50(2)
Pb1	Br1^1	3.0696(14)	C6	C7	1.36(3)

Br1	Pb1[2]	3.0695(14)	C6	C5	1.36(3)
N1	C1	1.527(17)	C4	C5	1.40(2)
C3	C1	1.501(19)	C8	C7	1.39(3)

[1]1/2+X,3/2-Y,1-Z; [2]-1/2+X,3/2-Y,1-Z

Table S6. Bond angles for *S*-$MBAPbBr_3$-293 K.

Atom	Atom	Atom	Angle/°	Atom	Atom	Atom	Angle/°
Br3	Pb1	Br1	93.50(4)	C8	C3	C4	118.5(14)
Br3	Pb1	Br1[1]	88.26(4)	C3	C1	N1	108.4(11)
Br2	Pb1	Br3	88.77(4)	C2	C1	N1	108.2(12)
Br2	Pb1	Br1	84.92(4)	C2	C1	C3	116.3(12)
Br2	Pb1	Br1[1]	82.84(4)	C5	C6	C7	118.6(18)
Br1	Pb1	Br1[1]	167.59(3)	C3	C4	C5	119.3(16)
Pb1	Br1	Pb1[2]	82.48(3)	C3	C8	C7	121.5(15)
C4	C3	C1	121.1(13)	C6	C7	C8	120.2(18)
C8	C3	C1	120.4(12)	C6	C5	C4	121.8(16)

[1]1/2+X,3/2-Y,1-Z; [2]-1/2+X,3/2-Y,1-Z

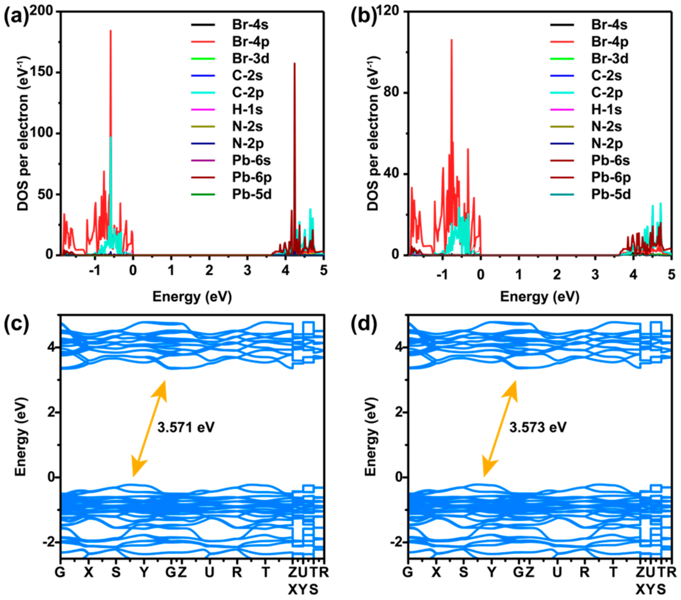

Figure S2. The electronic structures of $MBAPbBr_3$. (a-b) The PDOS of *R*-$MBAPbBr_3$ (a) and *S*-$MBAPbBr_3$ (b). (c-d) The band structures of *R*-$MBAPbBr_3$ (c) and *S*-$MBAPbBr_3$ (d).

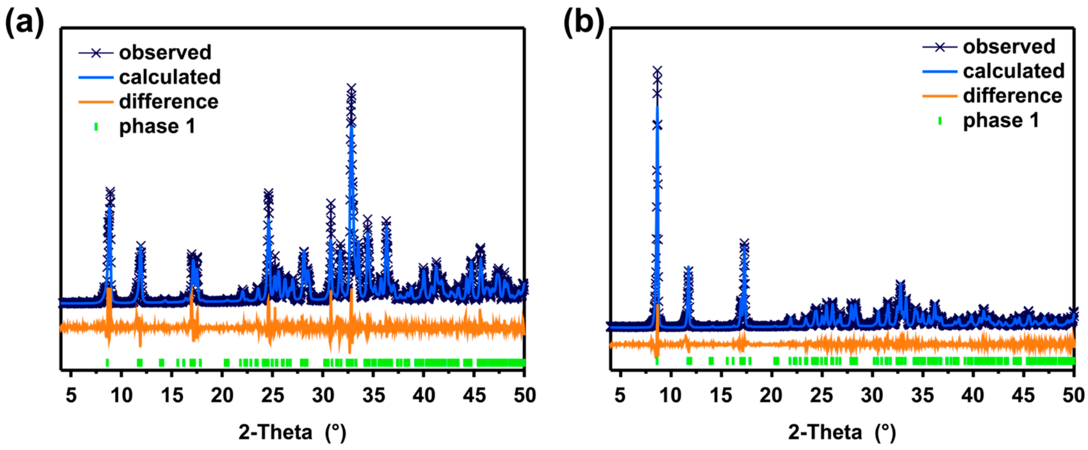

Figure S3. The PXRD fitting of *R*-$MBAPbBr_3$ (a) and *S*-$MBAPbBr_3$ (b).

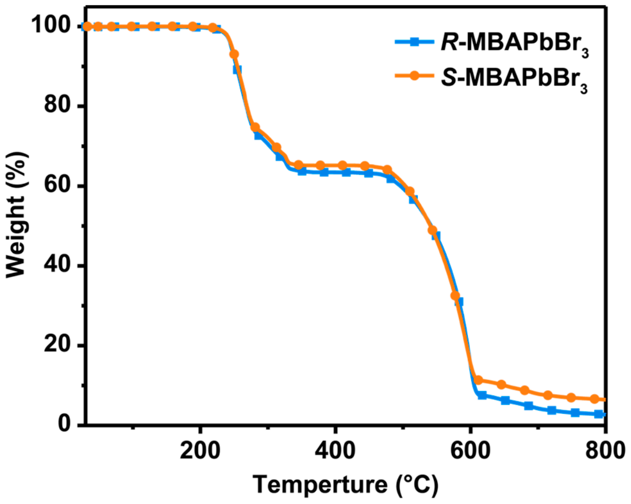

Figure S4. The TGA curves of *R*- and *S*-$MBAPbBr_3$.

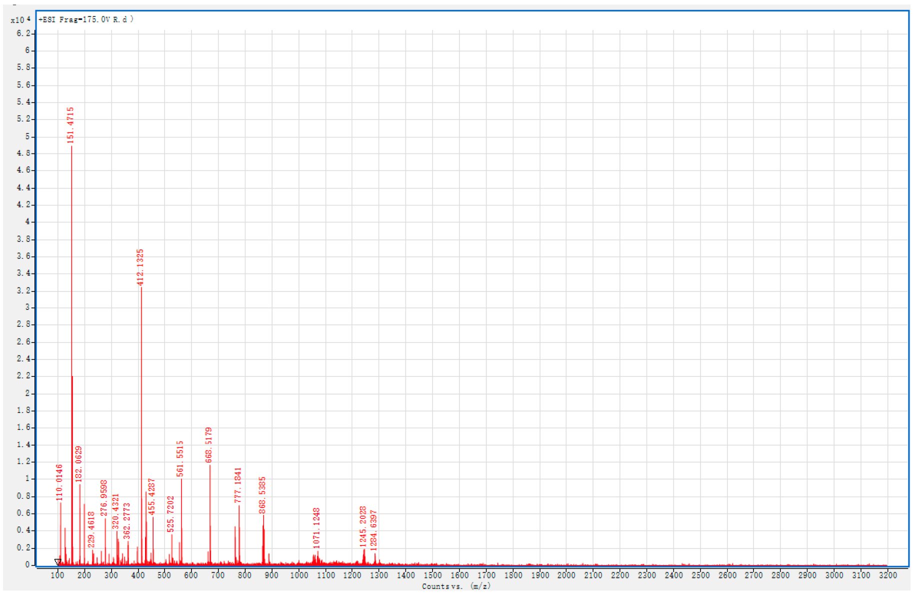

Figure S5. The mass spectrum (MS) of *R*-$MBAPbBr_3$.

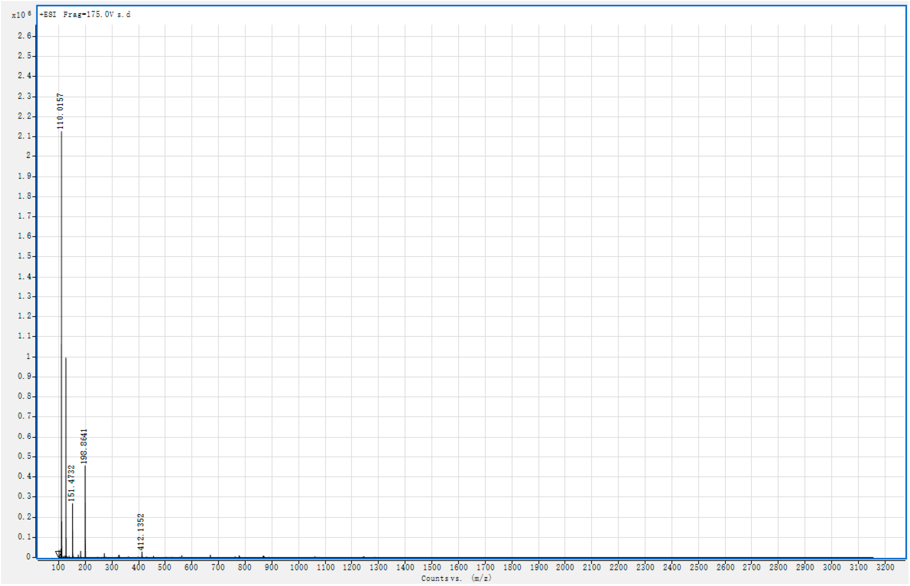

Figure S6. The mass spectrum (MS) of *S*-MBAPbBr$_3$.

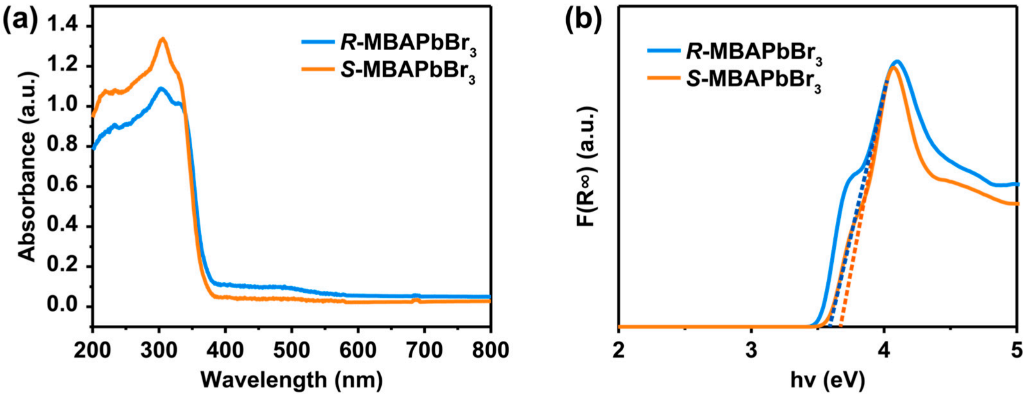

Figure S7. (a) The UV–Vis absorption spectra of MBAPbBr$_3$. (b) The band gap of MBAPbBr$_3$ fitted by the Kubelka–Munk function.

Table S7. Summary of the elastic properties of *S*-MBAPbBr$_3$. All the elastic tensors are obtained from DFT calculations. The maximal and minimal values of Young's modulus (E) and shear modulus (G) were determined by using the ELATE software. Anisotropy of X is denoted by $A_X = X_{max}/X_{min}$.

		S-MBAPbBr_3
G_{ij} (GPa)	C_{11}	20.04
	C_{22}	13.20
	C_{33}	20.98
	C_{44}	2.76
	C_{55}	19.46
	C_{66}	4.40
	C_{12}	3.31
	C_{13}	8.58
	C_{23}	-5.27
E (GPa)	$E_{\mathrm{max}}=E$<101>	33.2
	$E_{\mathrm{min}}=E$<011>	6.5
	A_E	5.1
G (GPa)	G_{max}=G(001)<010>	19.5
	$G_{\mathrm{min}}=G$(001)<100>	2.8
	A_G	7.0
Bulk modulus (GPa)		7.3
